# Supplementary material for: Anticipating the Novel Coronavirus Disease (COVID-19) Pandemic
Source: Front Public Health. 2020 Sep 3;8:569669. doi: 10.3389/fpubh.2020.569669 (PMC7494973; doi:10.3389/fpubh.2020.569669)
Supplement: Supplementary file 2 [file Data_Sheet_1.PDF]

Electronic Supplementary Material:

Anticipating the novel coronavirus disease (COVID-19) pandemic

1 Early Warning Indicators

Apart from the variance and the ACF(1), other known statistical indicators of critical slowing down, such as density ratio, skewness, and kurtosis can also unfold the ability to forewarn an upcoming epidemic transition [1]. Here, we investigate trends in these indicators in the proximity of a transition phase (high-burden disease state) in incidence curves associated with the COVID-19 outbreak, for each country studied in the main text. We analyse the potential of these indicators in paving their way in designing effective non-pharmaceutical interventions.

1.1 Density ratio

The phenomenon of critical slowing down can be revealed through changes in the correlation structure of a time-series distribution. We calculate short term correlation (at lag-1) of a time-series before the transition in the incidence curves, using ACF(1) in the main text. However, correlation structure at higher lags is ignored while calculating the lag-1 autocorrelation function of a time-series.

Here, we adopt power spectrum analysis to measure the long-term memory of a time-series [2]. Close to a transition phase, changes in the power spectrum can be associated with spectral reddening and thus leads to a shift in the power of spectral densities to low frequencies [2, 3, 4, 1]. Therefore, the density ratio, which is calculated as the spectral ratio of the spectral density at low-frequency to the spectral density at high-frequency [4], increases near a transition.

1.2 Skewness and Kurtosis

In the vicinity of two alternative stable states, slowing down can exhibit asymmetry in the probability distribution of a time-series [4, 1]. Skewness measures the degree of distortion of a time-series distribution from the symmetrical normal distribution curve. It is calculated as standardized third moment around the mean of a time-series distribution. Thus, vanishes for time-series symmetric about the mean and depicts asymmetry with positive/negative values. Positive skewness indicates fatter or longer tail on the right side of the distribution and vice-versa for the negative skewness.

Furthermore, decrease in the curvature of a time-series distribution due to occurrence of rare values can be estimated through Kurtosis. Particularly, it estimates the presence of outliers in the distribution. Near an upcoming transition, the time-series distribution may become leptokurtic [4, 1]. Thus, kurtosis reveals longer and fatter distribution which indicates profusion of outliers and is measured as standardized fourth moment around the mean. Both the skewness and the kurtosis are calculated as:

$$\text{Skewness} = \frac{\frac{1}{n}\sum_{t=1}^{n}(x_t-\mu)^3}{\sqrt{\frac{1}{n}\sum_{t=1}^{n}(x_t-\mu)^2}^{\,3}}, \tag{2.1}$$

$$\text{Kurtosis} = \frac{\frac{1}{n}\sum_{t=1}^{n}(x_t-\mu)^4}{\left(\sqrt{\frac{1}{n}\sum_{t=1}^{n}(x_t-\mu)^2}\right)^{2}}. \tag{2.2}$$

We observe that the statistical tools namely density ratio, skewness (Eq. 2.1) and kurtosis (Eq. 2.2) exhibit signals of slowing down for data-sets of most of the countries (Fig. 1). However, the strength of the signals may vary amongst the countries. As observed for the trends in the variance and ACF(1) (main text), Density ratio gives weak signals and no trend is observed in the skewness and kurtosis for the time-series data of India (Fig. 1, panel A). EWSs analysis on the data-sets of all countries, except Italy (Fig. 1, panel G) indicates an increase in the density ratio in the time-series distribution before the sudden rise in the curves (Fig. 1). While skewness and kurtosis alarm and upcoming rise for most countries investigated, the indicators of slowing down does not reveal an upcoming transition for South Korea (Fig. 1, panel C) and Italy (Fig. 1, panel G). In case of Singapore, density ratio and kurtosis show an increasing trend, however, skewness decreases in the time series distribution (Fig. 1, panel E). Overall, we find that investigation of generic indicators such as density ratio, skewness and kurtosis can be helpful in acquiring information of a transition beforehand, while the reliability of these tools may depend upon the data-sets investigated. In conclusion, with proper understanding, EWSs analysis can be used to assess clinical data-sets in case of resurgence of such epidemics and formulate control policies.

2 Sensitivity analysis

Sensitivity analysis reveals the importance of choice of combinations of window size and filtering bandwidth in the observed trends. Here, we study the impact of these two parameters on randomness in the observed estimates. We estimate the variance and ACF(1) using window sizes ranging from 40% to 90% of the time-series length and bandwidths ranging from 5% to 100% each with the increment of 1 point. Figure 2 reveals a contour plot of P values for different combinations of the rolling window size and filtering bandwidth for the set of 1000 surrogate time-series having same mean and variance as the original data-sets. We find that low probability to acquire the observed trends in the variance by chance for large window sizes and bandwidths, except for the dataset of South Korea (Fig. 2C) and Singapore (Fig. 2D), which doest not reveal strong increasing trend in the variance. While estimating ACF(1), we observe low significance in the trends for large bandwidths, that may over-fit the time series data. (Figs. 2A– 2G). However, in our work we choose bandwidths to avoid under-fit and over-fit. The analysis thus suggests that it is obligatory to have proper understanding of the effect of window sizes and bandwidths on the EWSs indicators.

3 Surrogate analysis

To test the plausibility of acquiring the trend statistics by chance, we generate 1000 surrogate data-sets in two different ways. In the main text the surrogate data-sets

generated follow similar distribution (mean and variance) as the original records and probability of having randomness in the trend statistics is provided in the Table 4. Here, we produce surrogate time-series that have same Fourier spectrum and amplitudes as the original data-sets [5, 6]. Particularly, we apply discrete Fourier transform operator to time-series $(x(t),\ t = t_o,\ t_1, ..., t_{n-1} = 0, \Delta t, ..., (n-1)\Delta t)$ to obtain

$$X(f) = \sum_{k=0}^{n-1} x(t_k) e^{2\pi \iota k f \Delta t}, \tag{2.3}$$

where $f = -n\Delta f/2, ..., -\Delta f, 0, \Delta f, ..., n\Delta f/2$ corresponds to different frequency components. Further Eq. 2.3 is expressed as

$$X(f) = \mathcal{A}(f) e^{\iota \phi(f)}, \tag{2.4}$$

having amplitudes $\mathcal{A}$ and phase $\phi(f)$. Further from Eq. 2.4, we generate a phase randomised Fourier transformed time-series [7]. Applying inverse Fourier transform to the generated phase randomised series, the surrogate time-series is produced with same power spectrum as original data-sets [5, 6].

We calculate the likelihood of randomness in the observed trend estimates as the fraction of the surrogate data having trend estimates equal to or higher than the statistics of the original time-series (i.e. $\mathrm{P}(\tau^* \leq \tau)$). We construct a histogram plot indicating density distribution of the trend estimates for the set of 1000 surrogates (Fig. 4). We observe that the trends in both the indicators (variance and ACF(1)) are highly significant for the studied data sets, however, for South Korea (Fig. 4B), Singapore (Fig. 4D) and UK (Fig. 4G), the likelihood of randomness is relatively high. Overall, our results are comparable to those observed in the main text (Fig. 4) indicating in general low probability of randomness in the trends estimated for both the variance and the ACF(1). The probability, to obtain the estimated trend statistics of original data by chance by generating surrogates of same Fourier spectrum and amplitudes is mentioned in the Table 2.

We conclude that, with respect to predict the incidence curves of epidemics, it is critically important that to capture signals of critical slowing down the system must be preceded gradual loss of resilience. EWSs may hint whether the system is entering a situation which may demand intensive public health resources and advices rather than buffering the disease spread. However, proper understanding of the EWSs tools and the selection of moving window size and Gaussian filtering bandwidth can pose further challenges and limitations.

4 Detection of the transition phase

We adopt a method that detects the abrupt shifts by analysing the gradient changes that may occur over a short period in the time series [8]. To search for the anomalous changes in the gradient of the time series, we consider the entire dataset of incidence curves, for each country. We separate the time series of length n into d-sized segments. Within each segment, we fit a linear regression and calculate the gradient. From the set of gradients we record the significant gradients, i.e gradients which are more than 3 median absolute deviations (MADs) away from the median. The value 1 is added/subtracted in the data points corresponding to the significant gradients, which are zero otherwise. We repeat the

above procedure for range of segment sizes d. The detection time series is thus obtained, which is divided by the number of d used. We plot the detection value and record the time of transition in each case (Fig. 5). Thus, for the EWSs analyses, we use the incidence curve dataset before the happening of the actual transition. We use R package "asdetect" [8] to detect the abrupt shift in the simulated population time series.

We find significant shifts occurring in the incidence curves from low burden to high burden state in most of the countries (see Fig. 5). However, significant change in the gradient is not detected for the datasets of Italy and Spain. Also, the degree of the shift varies amongst countries, where 1 depicts a high shift and 0 depicts no change in the gradient of the time series.

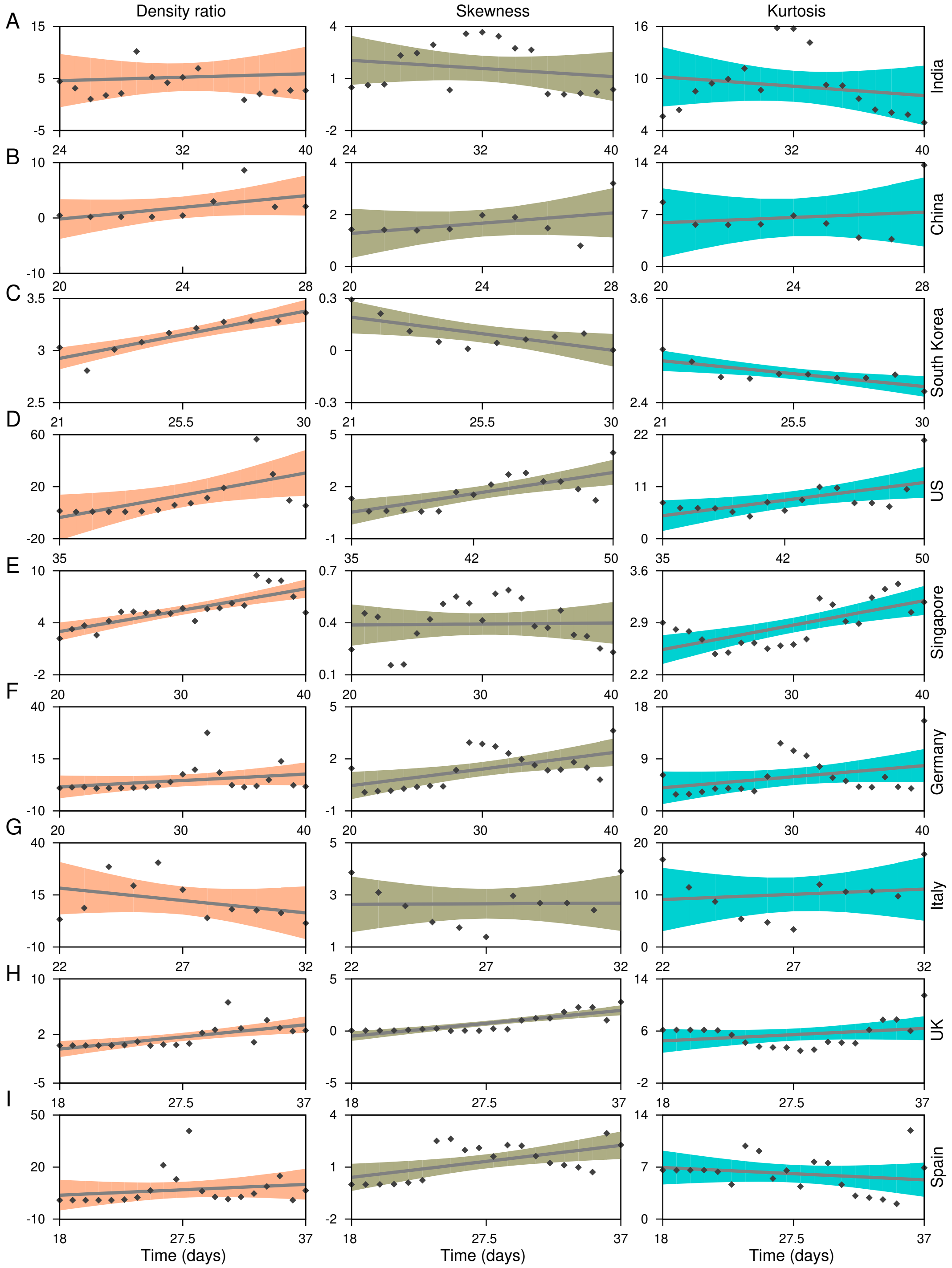

Figure 1. Statistical trends observed in the EWSs analysed to investigate forthcoming transition in the COVID-19 incidence curves. Panels depict the trends observed in the coefficient of variation (CV), density ratio, skewness and kurtosis in the data-sets of India (A), China (B), South Korea (C), US (D), Singapore (E), Germany (F) Italy (G), UK (H), and Spain (I). Scattered points are the estimated values of the respective slowing down indicators, where solid lines reflect the increasing/decreasing trend in the indicators and are obtained by fitting linear regression models. The shaded regions are the confidence bounds for the fitted models.

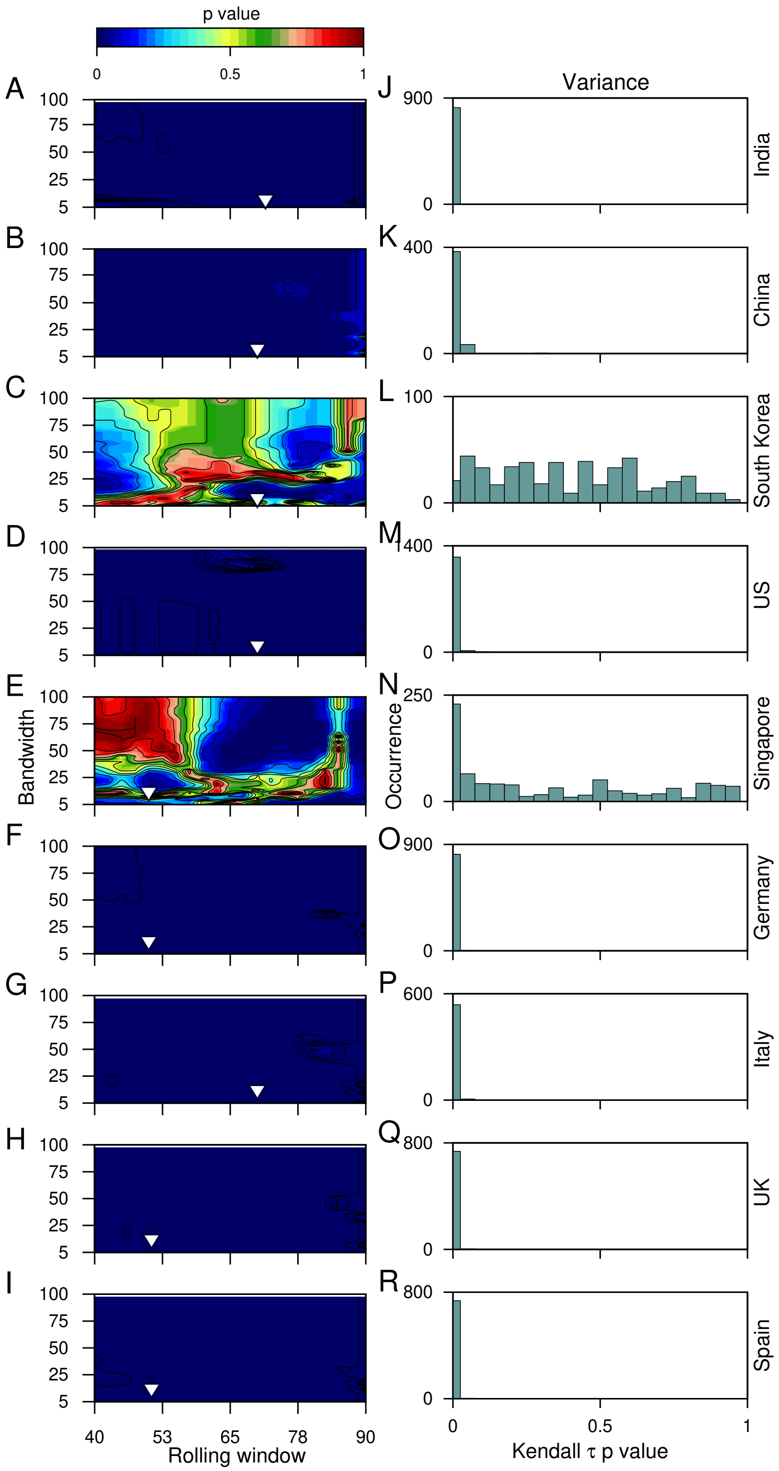

Figure 2. Significance test for the incidence curve data-sets using different combinations of moving window size and filtering bandwidth. A-I: Contour plots and J-R: histogram plots, of P values estimated from the test statistics of the surrogate time-series for the variance. The surrogate data-sets are generated by picking data with replacement from bootstrapped and shuffled the original residual time-series (Materials and Methods, main text).

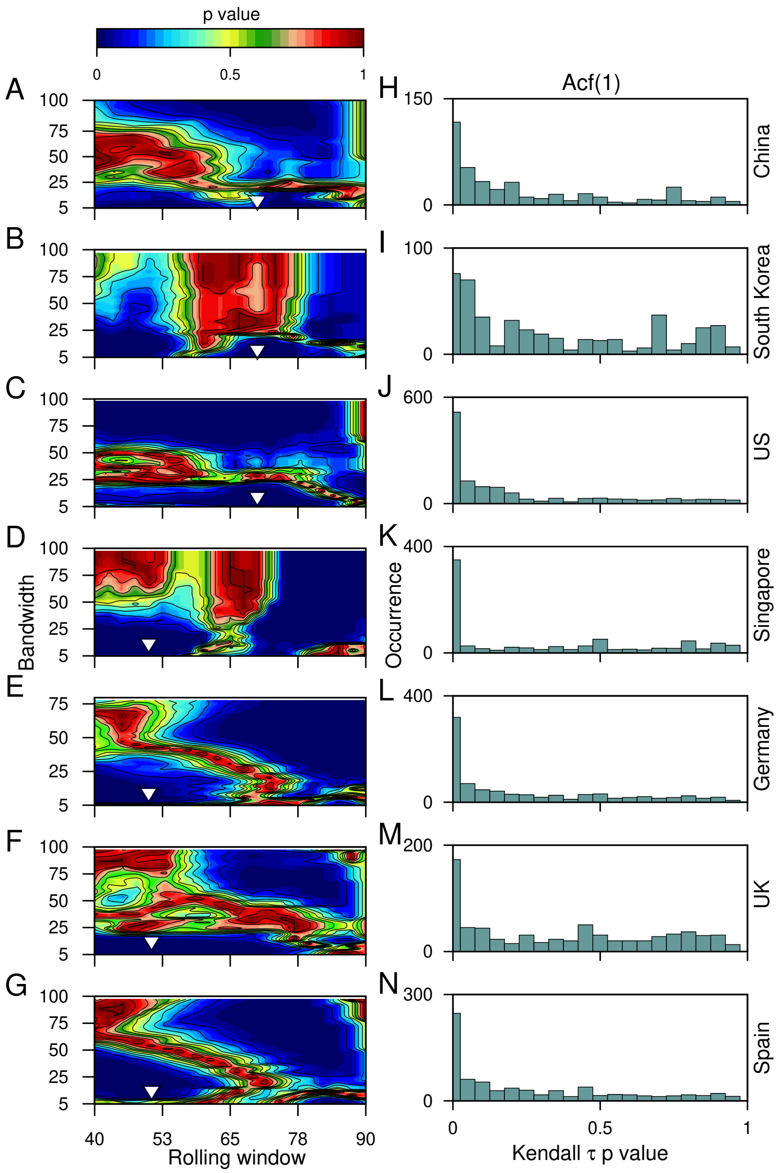

Figure 3. Significance test for the incidence curve data-sets using different combinations of moving window size and filtering bandwidth. A-G: Contour plots and H-N: histogram plots, of P values estimated from the test statistics of the surrogate time-series for the autocorrelation function at first lag. The surrogate data-sets have same Fourier spectrum and amplitudes as the original data-sets.

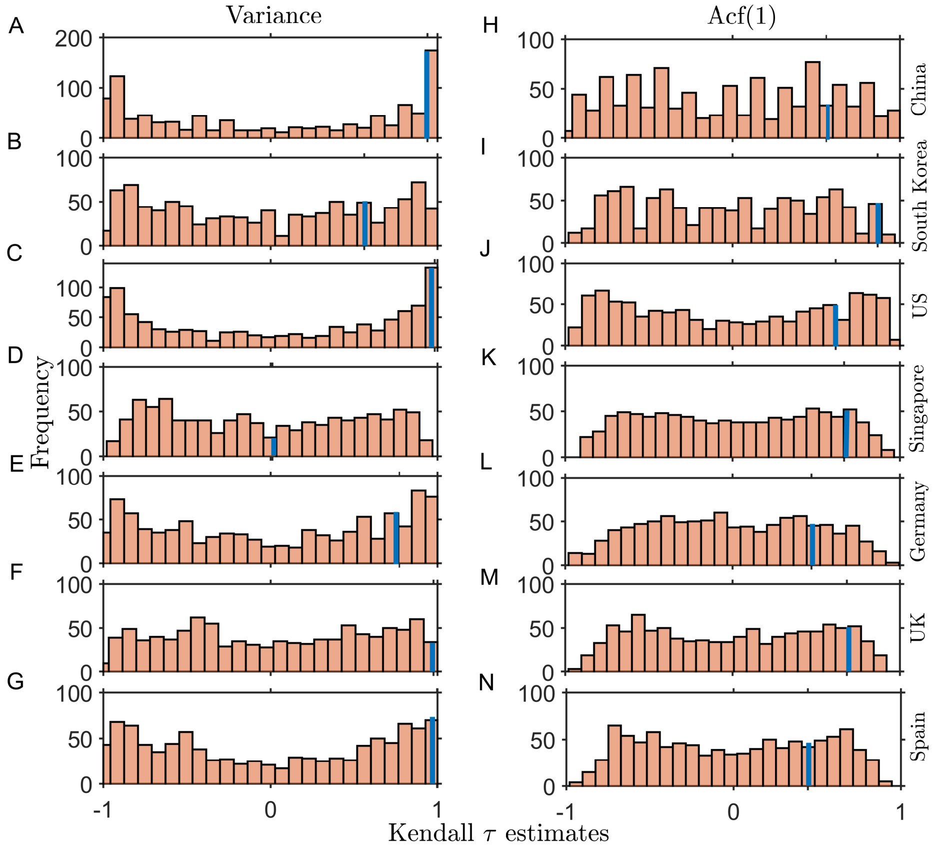

Figure 4. Histogram plots demonstrating the distribution of the Kendall-τ estimates obtained for the set of 1000 surrogate time-series having the same Fourier spectrum and amplitudes as the original time-series. The trend estimates are calculated in both the variance (left panels, A-G) and ACF(1) (right panels, H-N) for the countries studied in the main text. Solid lines indicate the limit beyond which the Kendall-τ of the surrogate data is higher than the statistic observed in the ACF(1) and variance of the original time-series.

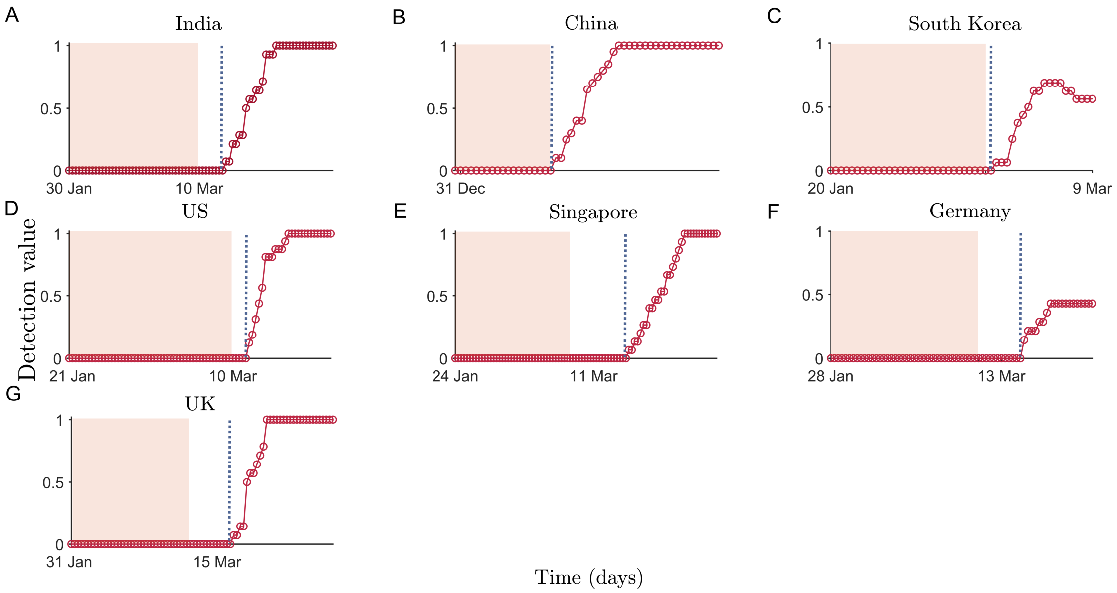

Figure 5. Detection of the onset of the transition phase in the data sets of each country analysed in the main text. A-I: Detection time series, where the dashed line depicts the anamolous change in gradient and onset of transition in the incidence curve. Shaded region depicts the dataset used to carry out EWSs analyses for each country.

Table 1. Country wise dates of the onset of restricted public movement or respective nation-wide lockdown [9], along with the fraction of infected cases reported at the mentioned dates. Note that the fraction of infected cases is calculated over the total population of each country, which is considered to be constant.

**Country**	**Lockdown dates**	**Fraction of the affected population during lockdown**
India	25 March	$\approx 2.36 \times 10^{-7}$
China	23 January	$\approx 4.47 \times 10^{-7}$
South Korea	Mid February (social distancing measures)	$\approx 3.09 \times 10^{-7}$
US	Nearly 25 March (partial lockdown)	More than 1.7×10^{-4}
Singapore	27 March (partial lockdown)	$\approx 9.89 \times 10^{-5}$
Germany	23 March	$\approx 2.98 \times 10^{-4}$
Italy	9 March	$\approx 1.22 \times 10^{-4}$
UK	23 March	$\approx 8.54 \times 10^{-5}$
Spain	14 March	More than 9.05×10^{-5}

Table 2. Probability of, by chance, obtaining the observed trend statistic of the original data for the set of 1000 surrogates having same Fourier spectrum and amplitudes as the original data-sets. The likelihood of randomness in the estimated variance and ACF(1) is mentioned for the data-sets of each country studied in the work.

**Country**	**Window size**	**Bandwidth**
India	70	12
China	70	12
South Korea	70	12
US	70	12
Singapore	50	12
Germany	50	12
Italy	70	12
UK	50	12
Spain	50	12

Table 3. Probability of, by chance, obtaining the observed trend statistic of the original data for the set of 1000 surrogates having same Fourier spectrum and amplitudes as the original data-sets. The likelihood of randomness in the estimated variance and ACF(1) is mentioned for the data-sets of each country studied in the work.

**Country**	**Kendall-τ (Variance)**	**Kendall-τ (ACF(1))**
China	0.17	0.16
South Korea	0.26	0.01
US	0.05	0.22
Singapore	0.47	0.11
Germany	0.19	0.19
UK	0.001	0.11
Spain	0.003	0.26

Movie 1. Spatial distribution of total column NO_2 for the period of our study.

References

[1] V. Dakos, S. R. Carpenter, W. A. Brock, A. M. Ellison, V. Guttal, A. R. Ives, S. Kéfi, V. Livina, D. A. Seekell, E. H. van Nes, and M. Scheffer. Methods for Detecting Early Warnings of Critical Transitions in Time Series Illustrated Using Simulated Ecological Data. *PLoS One*, 7:e41010, 2012.

[2] Thomas Kleinen, Hermann Held, and Gerhard Petschel-Held. The potential role of spectral properties in detecting thresholds in the earth system: application to the thermohaline circulation. *Ocean Dynamics*, 53(2):53–63, 2003.

[3] Manfred Schroeder. *Fractals, chaos, power laws: Minutes from an infinite paradise.* Courier Corporation, 2009.

[4] Reinette Biggs, Stephen R Carpenter, and William A Brock. Turning back from the brink: detecting an impending regime shift in time to avert it. *Proceedings of the National academy of Sciences U.S.A.*, 106(3):826–831, 2009.

[5] James Theiler, B Galdrikian, Andrée Longtin, Stephen Eubank, and J Doyne Farmer. Using surrogate data to detect nonlinearity in time series. Technical report, Los Alamos National Lab., NM (United States), 1991.

[6] James Theiler, B Galdrikian, Andr Longtin, S Eubank, and J Doyne Farmer. Testing for nonlinearity in time series: the method of surrogate data. Technical report, Los Alamos National Lab., NM (United States), 1991.

[7] Dean Prichard and James Theiler. Generating surrogate data for time series with several simultaneously measured variables. *Physical Review Letters*, 73(7):951, 1994.

[8] Chris A Boulton and Timothy M Lenton. A new method for detecting abrupt shifts in time series. *F1000Research*, 8(746):746, 2019.

[9] Curfews and lockdowns related to the 2019–20 coronavirus pandemic. `https://en.wikipedia.org/wiki/Curfews_and_lockdowns_related_to_the_2019-20_coronavirus_pandemic`, 2020.
